# Editorial: Reviews in surgical oncology

**DOI:** 10.3389/fonc.2023.1242530

**Published:** 2023-07-20

**Authors:** Giorgia Radi, Cesare Ruffolo, Andrea Laurenzi

**Affiliations:** ^1^ Hepatobiliary Surgery and Organ Transplantation, IRCCS Azienda Ospedaliero-Universitaria di Bologna, Bologna, Italy; ^2^ Chirurgia Generale 3, Azienda Ospedaliero-Universitaria di Padova, Padova, Italy

**Keywords:** surgical oncology, training, high volume center, surgery, tumors

In the next decades, the oncological impact will progressively increase. In fact, it’s expected that within 2035 there will be 23,9 million of new oncological patients and 14,6 million of death related to cancer, however the distribution of mortality will not be homogeneous with a major rate in less developed countries (1).

Almost 80% of oncological patients will require a surgical intervention that are estimated to be 45 million within 2030 (2).

It’s well known that most of oncological patients does not have an immediate access to a proper oncological surgery in high volume centers.

Although there are several reasons to this problem, one of the leading causes is the lack of surgeons properly trained in the management of oncological patients.

Surgical oncology is defined as a subspecialty of surgery applied to oncology, from diagnosis to treatment. It’s still under debate if surgical oncology could be considered as a specialty itself, but there is almost full agreement on the fact that a single surgeon could not be skilled in the management of all type of cancers.

The surgeon operates within multidisciplinary teams together with oncologists, radiologists, radiation oncologists, pathologists etc. to plan the best diagnostic and therapeutic project for each patient. There are several evidence that show how this approach can modify patients’ management and lead to better outcomes (3), moreover also high-volume hospitals have shown to lead to reduction in postoperative mortality and morbidity (4).

In surgical oncology, surgeons can act in different phases: diagnostic and staging performing biopsies; in the treatment either with curative intent removing organs affected by tumor or in the palliative treatment leading to tumor reduction volume for reduced quality of life or in the context of emerging complications (i.e. bleeding, perforations etc). Moreover, surgeons can also intervene in the setting of prevention removing organs and/or tissues at high risk of degeneration in patients with genetic mutations or in the reconstructive phase such as breast cancer.

From the surgical point of view there are three pillars in the surgical oncology: patients’ selection, minimally invasive surgery and quality of oncological resections.

Patients’ selection has a double meaning; the first is associated to check if the patient is fit in half to undergo surgical procedures due to an older population with different types of comorbidities, the second is if the patient really benefits from a surgical intervention or if a neoadjuvant treatment could be envisaged to improve long-term outcomes.

Minimally invasive surgery had a widespread impact on surgical oncology during the last two decades, improving post-operative outcomes with comparable oncological outcomes, allowing faster access to adjuvant treatment, and improving quality of life.

The quality of oncological resections, either minimally invasive or conventional, remains one of the main goals of surgical oncology, since complete excision of tumor with adequate lymph-nodes removal and without residual micro-macroscopic tumor foci is associated to better long-term outcomes.

Considering the importance of this discipline and the impact of cancer on population, it would be of paramount importance to develop a strong educational process that allows training in all different oncological fields, considering the differences between type of cancers, their incidence, and available resources.

There are several limits to the development of such model from the lack of consciousness of such disease to the shortage of proper personnel and facilities.

For this reason it’s our opinion that evidence based medicine with the use of reviews represents a useful tool to promote a homogeneous level in cancer management, sharing good clinical practice with an adequate level of training, trying to minimize the differences between different health regions.

## Author contributions

GR wrote the article. CR critical view and correction. AL conceived and wrote the article. All authors contributed to the article and approved the submitted version.
